# Epigenetic editing for autosomal dominant neurological disorders

**DOI:** 10.3389/fgeed.2024.1304110

**Published:** 2024-03-06

**Authors:** Jennifer J. Waldo, Julian A. N. M. Halmai, Kyle D. Fink

**Affiliations:** Neurology Department, Stem Cell Program and Gene Therapy Center, MIND Institute, UC Davis Health System, Sacramento, CA, United States

**Keywords:** epigenetics, autosomal dominant, CRISPR/Cas9, neurodenerative diseases, gene regulation

## Abstract

Epigenetics refers to the molecules and mechanisms that modify gene expression states without changing the nucleotide context. These modifications are what encode the cell state during differentiation or epigenetic memory in mitosis. Epigenetic modifications can alter gene expression by changing the chromatin architecture by altering the affinity for DNA to wrap around histone octamers, forming nucleosomes. The higher affinity the DNA has for the histones, the tighter it will wrap and therefore induce a heterochromatin state, silencing gene expression. Several groups have shown the ability to harness the cell’s natural epigenetic modification pathways to engineer proteins that can induce changes in epigenetics and consequently regulate gene expression. Therefore, epigenetic modification can be used to target and treat disorders through the modification of endogenous gene expression. The use of epigenetic modifications may prove an effective path towards regulating gene expression to potentially correct or cure genetic disorders.

## Introduction

Epigenetics refers to the molecules and mechanisms that modify gene expression states without changing the nucleotide context (Cavalli and Heard, 2019). These modifications are what encode the cell state during differentiation or epigenetic memory in mitosis. Epigenetic modifications can alter gene expression by changing the chromatin architecture by altering the affinity for DNA to wrap around histone octamers, forming nucleosomes. The higher affinity the DNA has for the histones, the tighter it will wrap and therefore induce a heterochromatin state, silencing gene expression. Several groups have shown the ability to harness the cell’s natural epigenetic modification pathways to engineer proteins that can induce changes in epigenetics and consequently regulate gene expression (Jaenisch and Bird, 2003). Therefore, epigenetic modification can be used to target and treat disorders through the modification of endogenous gene expression (Rittiner et al., 2022).

This contrasts with traditional gene therapy and gene targeting approaches for the treatment of genetic disorders. Traditional gene therapy, using a viral vector such as adeno-associated virus (AAV) to deliver a gene for autosomal recessive disorders, has seen several successes including in spinal muscular atrophy (SMA1), Leber’s congenital amaurosis (RPE65) and AADC deficiency (Bainbridge et al., 2015; Chien et al., 2017; Mendell et al., 2017). While this has shown a lot of promise, it is incompatible with many disorders, as gene dosage is highly regulated within the cell and overexpressing certain genes could cause negative effects, as is seen in MECP2 duplication syndrome (Ramocki et al., 2010). It is also not possible for diseases caused by gain-of-function mutations, as gene replacement strategies would be rendered ineffective in the presence of a dominant negative protein and could even exacerbate pathophysiology. Antisense oligonucleotides (ASO) and RNA interference (RNAi) have shown promise, with approved therapies for Spinal Muscular Atrophy (Nusinersen/Spinraza) and Duchenne Muscular Dystrophy (Eteplirsen), as well as therapies in late-stage clinical trial such as transthyretin-mediated amyloid polyneuropathy (ATTRv-PN), but these therapeutics only knock down at the RNA level. This makes RNAi and ASOs generally unable to fully abolish gene expression and may have lower efficacy in disorders where RNA toxicity is observed (White et al., 2012; Heinz et al., 2021). This presents the aspect of knockdown at the DNA level as increasingly attractive as it can achieve high levels of downregulation along with limiting RNA toxicity.

Neurological disorders are the number one cause of disability and the second leading cause of death worldwide and have a large unmet need when it comes to therapeutic creation (Feigin et al., 2019). While there are over 600 known neurological diseases, only a subset of cases have a known genetic cause (US National Library of Medicine). The advent of new sequencing methodologies and decreasing costs have allowed for the identification of an increasing number of genetically linked neurological disorders, but the complexity and rarity of some neurologic disorders continue to be a barrier to therapeutic production (Bras et al., 2012). Additionally, neurological disorders are particularly challenging to treat due to the combined nature of the complexity of the brain, diverse cell types, lack of tissue regeneration, and obstacles to therapeutic entry such as the blood-brain barrier. Timing of the treatment is also an important factor to consider, as many neurodevelopmental disorders show changes in brain formation *in utero* and neuronal plasticity decreases as we age. This makes the ability to retain or improve neurological function in neurodegenerative disorders variable based on how early the treatment can be administered. Approval rates for CNS targeting therapeutics are lower than those for other parts of the body and most do not alter the progression of disease or address the underlying problem and focus more on treating symptoms (Kesselheim et al., 2015). This necessitates the need for novel therapeutic avenues to try and address the large unmet need.

There has been great success using gene editing, such as CRISPR-Cas9, within the clinic for some hematological disorders. *Ex vivo* gene editing, where the cells of interest are edited outside of the patient and then returned, has shown great promise in the treatment of sickle cell, functionally curing patients (Frangoul et al., 2021). These *ex vivo* therapies are challenging with an organ such as the brain, as neuronal cells are particularly sensitive and are unlikely to survive surgical removal, gene correction and transplantation into the brain, along with the complex neuronal circuitry that would be disrupted during the highly invasive procedure (Gowing et al., 2017). While *ex vivo* gene therapy is not applicable to neurological disorders, it shows the clinical feasibility and applicability of gene editing for human disease. Epigenetic modification allows for tunable modification of gene expression while still maintaining the endogenous genome structure and regulation that naturally occurs within the cell. The field of directed epigenetic modification has been rapidly evolving with the advent of DNA binding domains such as TALEs, Zinc Fingers, and CRISPR-dCas9. This presents a novel therapeutic avenue that could be very impactful in the field of dominant neurological disorders.

## Dominant disorder pathology

**FIGURE 1:**
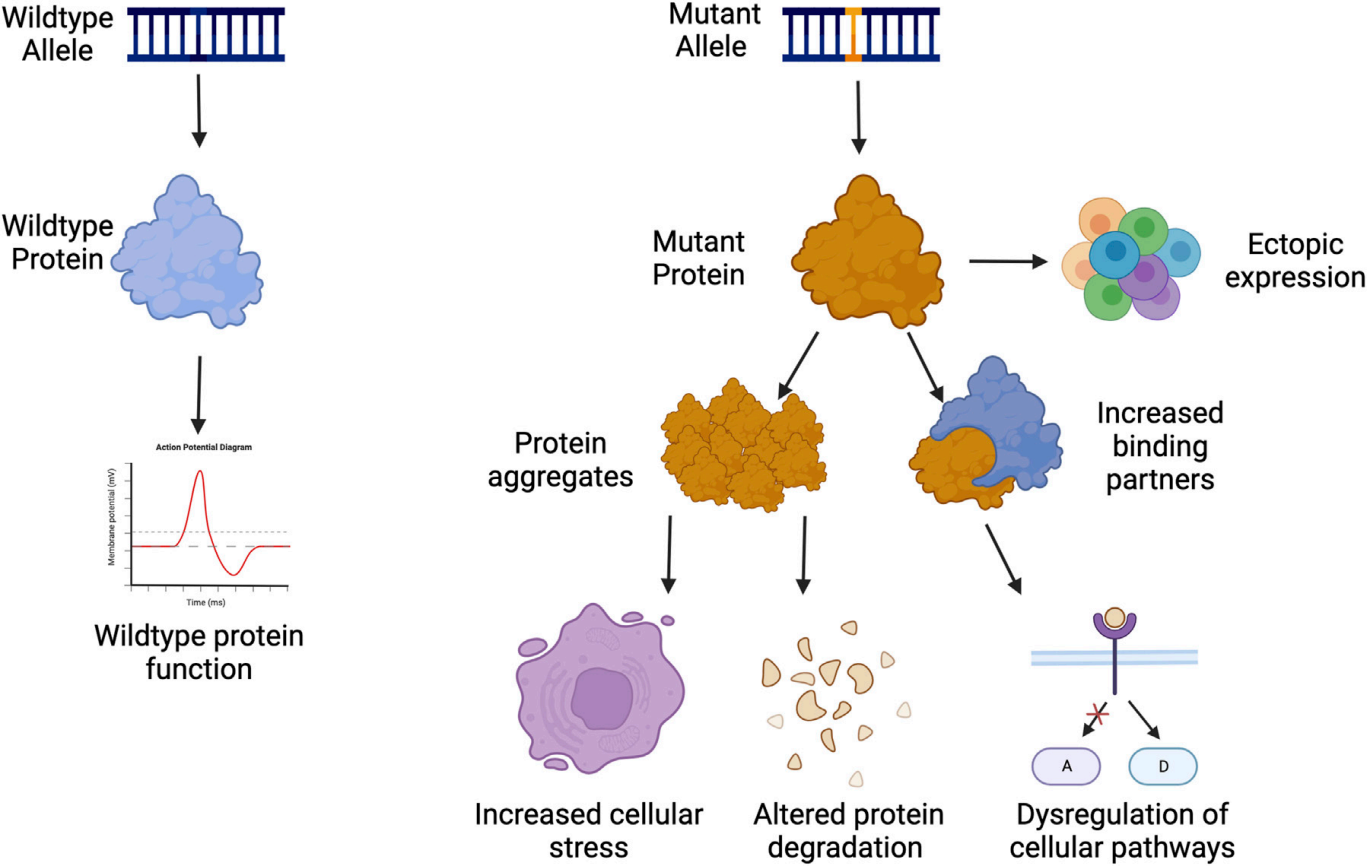
Examples of gain of function pathogenesis. Created with Biorender.com.

Dominant negative disorders are usually rare diseases caused by a mutation in a single gene resulting in a gain-of-function in the protein. This phenomenon was originally termed antimorph by Muller, and later as a dominant negative mutation by Ira Herskovitz as a “mutant polypeptides that when over-expressed disrupt the activity of the wild-type gene” (FlyBase Reference Report, 1932; Herskowitz, 1987). These gain-of-function (GOF) mutations often occur on protein interaction domains, which either result in ectopic expression, blocking of healthy protein function, increased activity of the mutant product, or novel activity that the protein did not previously perform (Figure 1) (Backwell and Marsh, 2022; Gerasimavicius et al., 2022). Occasionally, the increased activity can lead to an increased number of binding partners, which can dysregulate or activate multiple pathways in the cell that can lead to pathogenesis (Figure 1) (Ségalat, 2007; Backwell and Marsh, 2022; Gerasimavicius et al., 2022).

GOF disorders present a unique challenge towards treatment development, as traditional gene therapies of gene supplementation are not possible as they are with many loss-of-function or haploinsufficient mutations. GOF proteins are often a part of homodimers, heterodimers, or protein complexes making the addition of more functional proteins ineffective as the mutated protein can sequester the functional copy. This can in turn make the amount of functional protein below 50% as expected from a heterozygous mutation. The addition of more healthy proteins could exacerbate pathogenesis by becoming sequestered in protein aggregates, which can affect protein clearance and degradation throughout the cell (Figure 1).

Nucleotide repeat disorders are of particular interest when discussing dominant neurological disorders, as there are currently 20 disorders caused by unstable nucleotide repeats that include both loss of function and gain of function proteins. Many dominant disorders are caused by trinucleotide repeats, particularly those that affect neurologic function. Genes implicated in trinucleotide repeat disorders are often highly expressed in the brain and lead to neurodevelopmental or neurodegenerative phenotypes. Genes that contain a trinucleotide repeat are prone to expansion due to the DNA secondary structures that can form during DNA replication causing a slippage of the DNA polymerase and further expansion of the repeat (McMurray, 2010). It is particularly common for these to be CAG repeats that encode for a polyglutamine tract. This increased polyglutamine tract creates an insoluble protein, which increases its pathogenesis and its ability to form toxic aggregates that can then impair the protein clearance system of the entire cell (Ciechanover and Brundin, 2003). Many of these toxic proteins are also localized to the nucleus, where they can interact with transcription factors such as CREB-bind protein (CBP), p53, and TATA box binding protein (TBP) and induce global impairment or changes to the transcriptome (McCampbell et al., 2000; Steffan et al., 2000; Schaffar et al., 2004). These polyglutamine proteins are also seen to dysregulate cytoplasmic functions such as axonal transport and mitochondrial function, impairing the signaling and energy production within the cell, making them particularly detrimental in neuronal cells (Panov et al., 2002; Gunawardena et al., 2003).

These genes themselves are also difficult to target, as trying to decrease the repeat itself can prove to be challenging as distinguishing between alleles is often not possible, nor is distinguishing the length of the repeat being targeted. Current approaches for repeat disorders such as Huntington’s Disease aim to either create indels to reduce *HTT* expression, or to completely remove the CAG repeat (Yang et al., 2017; Ekman et al., 2019). While this approach has shown promise, it may not be clinically feasible. Targeting a repetitive sequence of DNA precisely is difficult and can cause many off-target mutations (Ikeda et al., 2020). There are 9 different diseases caused by CAG repeats, as well as over 1,000 CAG arrays within the genome (Zeitler et al., 2019). This makes direct repeat targeting an unfavorable approach for the treatment of many disorders.

## Gene editing platforms

Gene editing has become an increasingly useful tool in the field of biology, whether for creating model systems, testing gene function, or for therapeutic development. Gene editing platforms have the commonality of having an innate DNA binding domain that can target specific regions of interest, along with an innate or fused nuclease domain to induce double-stranded breaks (DSBs). These DSBs are then resolved through non-homologous end joining (NHEJ) or homology-directed repair (HDR) to either introduce small insertions and deletions, large deletions, or introduce new fragments of DNA. Zinc fingers, TALE nucleases, and CRISPR-Cas9 are commonly used for these directed gene editing systems.

### Zinc fingers

**FIGURE 2:**
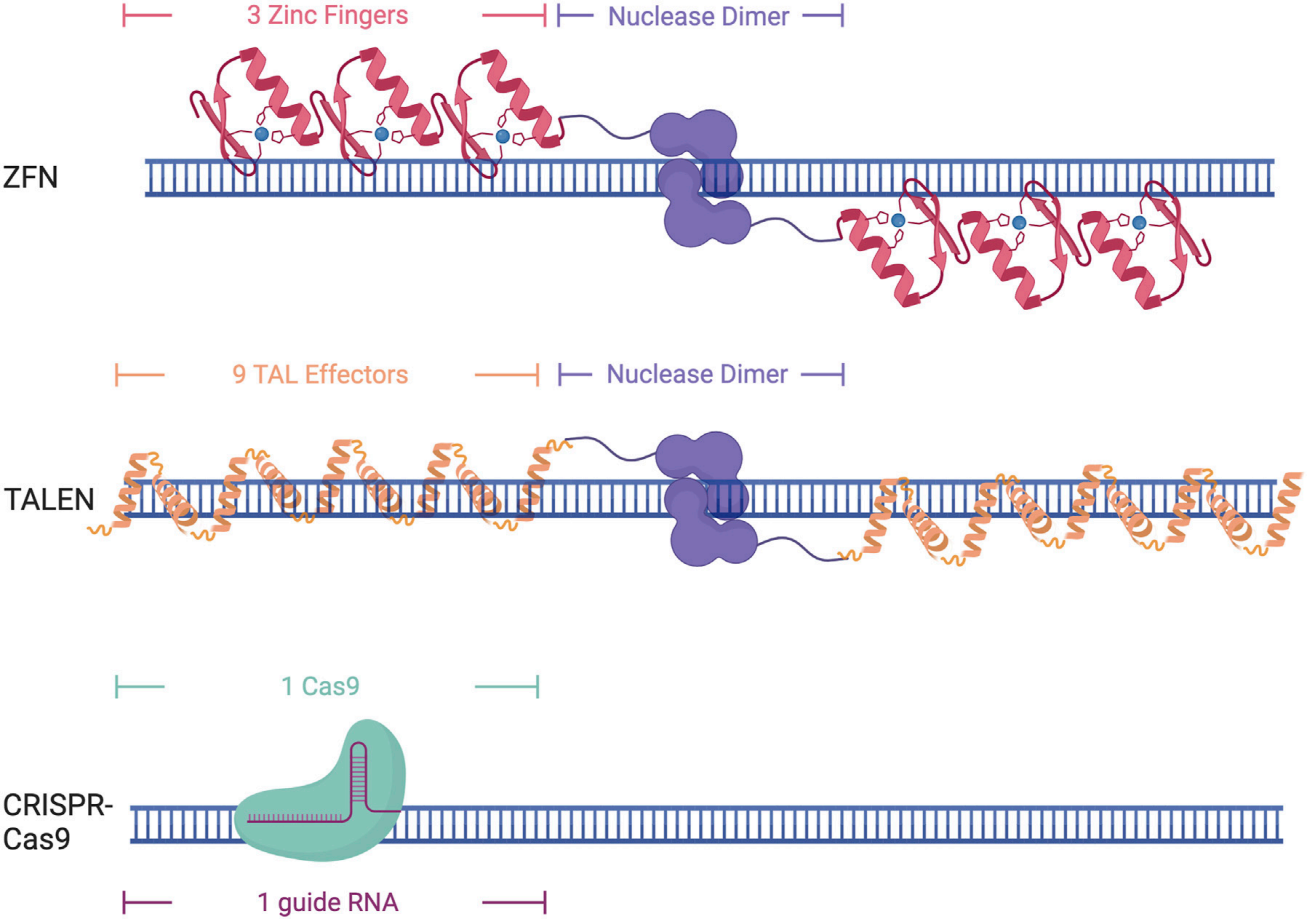
Different DNA-binding domains and their DNA targeting structure. Created with Biorender.com.

Zinc fingers are eukaryotic DNA binding domains comprised of two β-sheets and one α-helix in which the residue composition confers specificity (Miller et al., 1985; Pavletich and Pabo, 1991). Zinc fingers recognize 3 base pairs each, requiring the use of at least 6 Zinc Finger proteins tethered to make a larger DNA binding protein to target a specific genomic locus (Figure 2) (Miller et al., 1985; Pavletich and Pabo, 1991). As multiple zinc fingers are needed to target a specific locus, an entirely new protein needs to be synthesized for each nucleotide change, which can be tedious and difficult. As these proteins do not contain an endonuclease domain, one must be fused to them to induce DNA cleavage, such as Fok1. This necessitates the need for a pair of 6 zinc fingers to induce a double-stranded break, as Fok1 is a nickase and only cuts one strand of the DNA.

### Transcription activator like effectors (TALEs)

Transcription Activator Like Effectors (TALEs) were originally discovered in the plant pathogen *Xanthomonas pathovars* (Bonas et al., 1989). TALEs are large proteins that contain repeat domains that are 34 amino acids in length. Target specificity is conferred through the presence of two repeat-variable diresidues in positions 12 and 13 that allow them to recognize a single nucleotide. This makes them easier to design, as one base pair can be targeted at a time, although it still requires the synthesis of an entirely new protein for each single nucleotide change. This constitutes the need for upwards of 18 TALE domains to ensure specificity (Figure 2). Similar to Zinc Fingers, TALEs also lack endonuclease activity and require the addition of Fok1 to induce DNA cleavage.

### CRISPR-Cas

Clustered regularly interspaced short palindromic repeats (CRISPR) and its associated protein (Cas) were initially discovered as a part of the bacterial immune system to protect against viral infection (Bolotin et al., 2005; Mojica et al., 2005; Pourcel et al., 2005; Garneau et al., 2010). The Cas9 protein can bind to a specific region of DNA through sequence complementarity with a 17–20 bp single guide RNA (sgRNA) (Fu et al., 2014) (Figure 2). This sgRNA contains both the tracer RNA (tracrRNA) that recruits the Cas complex as well as the CRISPR RNA (crRNA) that is complimentary to the DNA sequence of interest (Karvelis et al., 2013). The only requirement for Cas9 binding and recognition of a locus is the recognition of a protospacer-adjacent motif (PAM) site, which varies by Cas protein being used, but is generally a 3-5bp sequence. The recognition of the PAM site allows stable R-loop formation and directs Cas9 nuclease to cut the DNA 3-5 base pairs upstream (Anders et al., 2014; Nishimasu et al., 2014; Sternberg et al., 2014; Palermo et al., 2017). While the need for an appropriate PAM site can diminish the targeting capabilities of CRISPR-Cas9, the discovery of new CRISPR-Cas proteins from different bacterial species and lab-evolved variants has greatly increased the applicability of this technology as they have different PAM recognition motifs or have virtually no necessary motif at all, allowing for the design of sgRNAs for almost any region of the genome (Friedland et al., 2015; Hu et al., 2018; Walton et al., 2020). Many of these evolved Cas9 variants have also been developed to enhance specificity (Slaymaker et al., 2016; Hu et al., 2018). This makes the CRISPR system an attractive choice for genomic studies as the ease of changing the sgRNA allows for efficient targeting of multiple loci in the genome and is much easier than protein-based DNA recognition that occurs with zinc fingers and TALEs. While Zinc fingers and TALEs both need a nuclease domain fused to them to induce double-stranded breaks, Cas9 has two nuclease domains, RuvC and HNH, that can induce double-stranded breaks through the binding of a single Cas9. Cas9 can be further modified to have a point mutation in one of the nuclease domains to create a nickase Cas9 (nCas9) that can be used to cut only one strand of the DNA (Ran et al., 2013). The creation of these nickases has also led to other types of Cas9 editing, including the ability to edit an individual base through the addition of cytidine deaminase or adenosine deaminase, or prime editing through the addition of a reverse transcriptase, allowing for small point edits to larger insertion or edits up to several hundred base pairs (Rees and Liu, 2018; Anzalone et al., 2019; Zheng et al., 2023).

While DNA binding domains work well for nucleotide edits to the genome, they induce double-stranded or single-stranded breaks, which can have many adverse effects such as selecting for cells that have an inactive tumor suppressor p53 pathway, or unintended genomic alterations such as single base pair indels or larger alterations such as translocations, duplications, or a loss of heterozygosity (Bouaoun et al., 2016; Ihry et al., 2018; Kosicki et al., 2018; Cullot et al., 2019; Rayner et al., 2019; Enache et al., 2020; Boutin et al., 2021; Leibowitz et al., 2021; Liu et al., 2021; Turchiano et al., 2021; Boutin et al., 2022; Höijer et al., 2022; Kosicki et al., 2022). Recent studies have also shown the genotoxic effects of using nickase-based systems, such as base editors or prime editors (Fiumara et al., 2023). While these effects have been mitigated by protein engineering, prior to clinical utility, safety concerns of customizable genome editors need to be carefully addressed (Yin et al., 2022a; Yin et al., 2022b; Yoo et al., 2022; Doman et al., 2023; Liao et al., 2023). This has not stopped the movement of base editors to the clinic, with Beam Therapeutics CAR-T cell therapy for relapsed, refractory lymphoma having dosed its first patient in 2023 (Clinical Trail ID NCT05885464). In addition, the FDA has now approved the first gene therapy for Cas9 gene editing for Sickle Cell Disease and Beta Thalassemia (Casgevy/Vertex Pharmaceuticals). It is important to note that both approaches require *ex vivo* editing of autologous CD34^+^ cells allowing for a vast *in vitro* safety assessment including off-target profiling highlighting further complexities of direct *in vivo* editing approaches (Frangoul et al., 2021). Base editing has also been applied for *in vivo* gene therapy in the liver for heterozygous familial hypercholesterolemia (HeFH), atherosclerotic cardiovascular disease (ASCVD), and uncontrolled hypercholesterolemia through VERVE-101(Clinical Trail ID NCT05398029). Prime editors have yet to make it to the clinic but are being heavily used in a multitude of genetic disorders. Although DNA editors such as Cas9, base editors, and prime editors have shown great efficacy, the possible side effects and mechanism of action necessitate the need for other pathways to therapeutic creation.

Recently, these DNA binding domains have been modified to regulate gene expression through epigenetics and chromatin remodeling.

## Epigenetic editing for transcriptional regulation

Gene expression is a tightly regulated process within the cell using both *cis* and *trans*-regulatory elements to ensure proper cell type gene expression and gene dosage. Epigenetic marks are strongly associated with gene expression and range from DNA methylation and histone tail modifications to large chromatin looping. The impact of such epigenetic marks is well characterized in cis-regulatory elements, such as proximal promoters or more distal enhancers (Shlyueva et al., 2014). DNA methylation is thought to be relatively mitotically stable and is enriched in the promoters of silenced genes, whereas it is seen to be enriched in the gene body of expressed genes (Yang et al., 2014). DNA methylation can also have a large effect when targeted to specific transcription factor binding sites, as many are methylation-sensitive, and their effect is ablated when they lose binding affinity via DNA methylation (Moore et al., 2013). Histone tails can incur many post-translational modifications (PTMs), ranging from methylation and acetylation to phosphorylation and ubiquitination (Huang et al., 2014). While promoters are generally marked by H3K4me3 and enhancers by H3K4me1, many histone modifications are marks of similar activation states for both regions (Sha et al., 2020). H3K27ac is a mark of active promoters or enhancers, and H3K27me3 is generally a mark of silenced or repressed promoters or enhancers (Dong and Weng, 2013). H3K9me3 is associated with transcriptionally silenced heterochromatic regions. H3K79me3 is often found in the body of actively transcribed genes as well as active enhancers. It is important to note that it is a matter of debate if all histone PTMs are mechanistically involved in transcription. The effect on gene expression due to histone tail modifications is not fully elucidated, with the possibility that their deposition does not change gene expression itself and is a consequence of the change in expression (Millán-Zambrano et al., 2022). Several groups have shown that for certain marks, such as H3K27ac, the targeted deposition can change gene expression (Mendenhall et al., 2013; O’Geen et al., 2017; Li et al., 2020). For other marks, such as H3Kme1/2/3 or H3K9me1/2/3, it is not directly known if their deposition alone can modulate gene expression (O’Geen et al., 2019).

It is also well understood that bivalent domains, such as those that contain both marks that maintain repression such as H3K27me3, and marks associated with activation such as H3K4me3, are often near genes that are poised for transcriptional activation (Dong and Weng, 2013). These bivalent domains are often changed based on cell state, such as upregulation of pro-neuronal genes upon differentiation from an embryonic stem cell, showing the ability for histone marks to predict gene expression changes throughout the life of a single cell (Dong and Weng, 2013).

The proteins that affect epigenetics within the cell are generally described in 3 categories: writers, readers, and erasers. Writers are enzymes that add chemical modifications, such as DNA methyltransferases, histone lysine methyltransferases, and histone acetyltransferases. Readers are proteins that bind to these chemical modifications to mediate their effects, such as DNA methylation readers, histone methylation, and acetylation readers as well as others. Erasers work to remove modifications that were laid down by writers, such as DNA demethylases as well as histone demethylases and histone deacetylases. Readers, writers, and erasers work throughout the genome to ensure proper gene expression and the proteins involved can be harnessed to modify gene expression in a targeted fashion.

These epigenetic regulatory proteins can be used as effector domains, where they are fused to DNA binding proteins to allow for targeted epigenetic modifications for the regulation of gene expression. To use Cas9 as an epigenetic editor, it must be rendered nuclease deficient through two point mutations in each of its catalytic domains, termed dCas9 (Qi et al., 2013). TALEs and zinc fingers have no natural nuclease activity and require no additional modification to be used as an epigenetic editor. Once these proteins are nuclease deficient, they are fused to effector proteins, which can induce changes in gene expression through the recruitment of transcription factors or chromatin remodelers to the genomic loci of interest.

**FIGURE 3:**
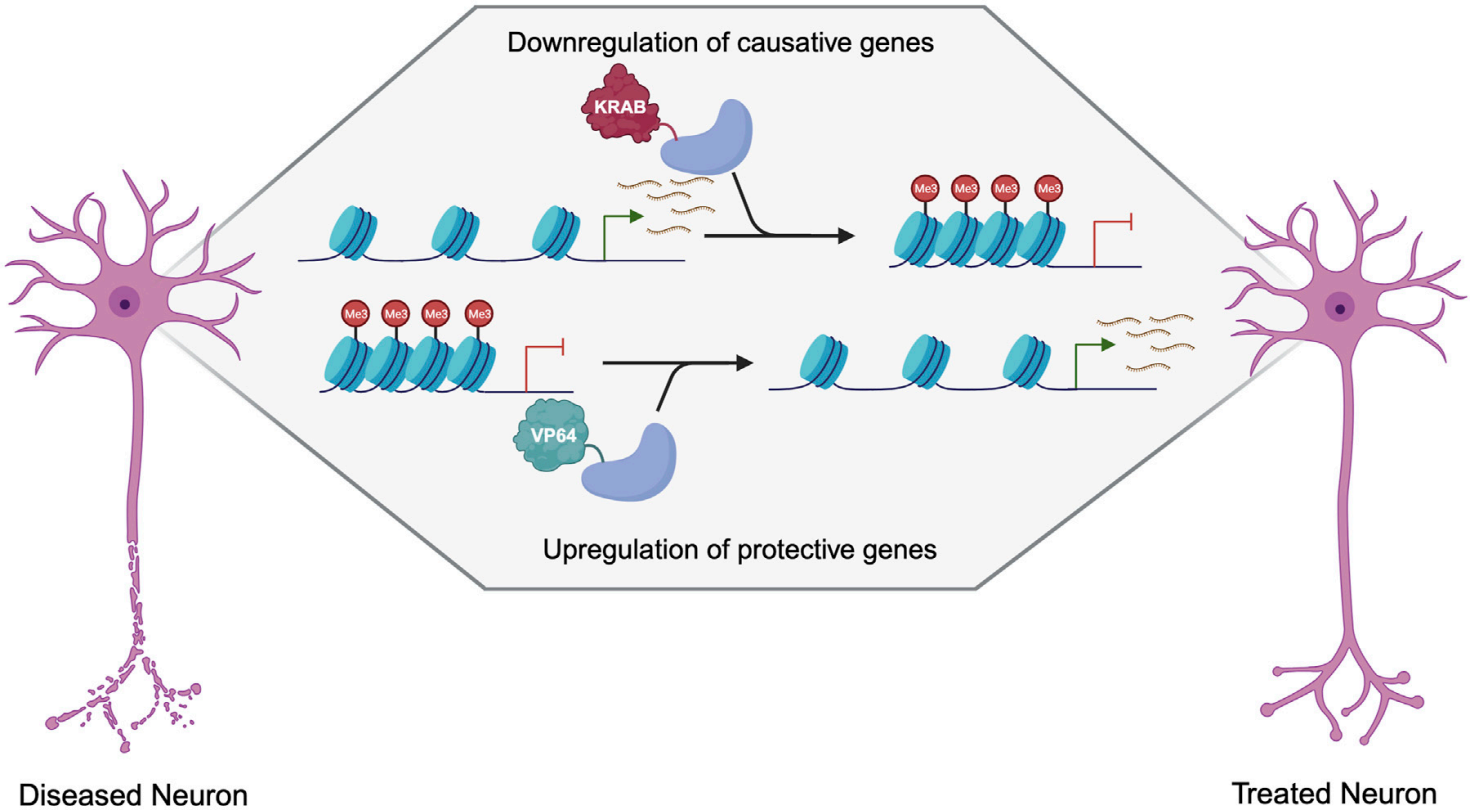
Therapeutic approaches to the treatment of neurogenerative disorders using dCas9 epigenetic editors. Created with Biorender.com.

Gain-of-function disorders present a unique opportunity for epigenetic editing as the downregulation of a mutant gain-of-function protein is likely to ameliorate many disease symptoms and pathogenesis (Figure 3). Initial studies showing downregulation using epigenetic editors showed that TALEs or zinc fingers fused to a KRAB effector domain were able to deposit H3K9me3 for robust knockdown (Witzgall et al., 1994; Tan et al., 2003; Zhang et al., 2013). While KRAB is still widely used for downregulation, many other effector domains can be used, as the effect of each effector is heavily dependent on the loci targeted (O’Geen et al., 2019). Several other effectors, such as Ezh2 and Fog1, deposit H3K27me3 for gene repression (Hong et al., 2005; O’Geen et al., 2017). Effector domains such as LSD1 may be useful when targeting distal regulator elements, as it removes H3K4me2 and H3K27ac, only when targeted to enhancers (Mendenhall et al., 2013). DNA methylation is also a prominent way to downregulate gene expression, as it is thought to be more heritable and stable than histone modifications. *De novo* methyltransferase 3A (DNMT3A) and its associated protein DNMT3L are often used to induce DNA methylation when fused to a DNA binding domain. This has been used with both TALEs and CRISPR-dCas9 to induce potent downregulation at specific loci (Bernstein et al., 2015; Stepper et al., 2017). A recent breakthrough in the field of epigenetic silencing is the ability to show persistent downregulation using the CRISPR-Cas9 system. Several groups have shown the ability to silence genes for more than 21 days in rapidly dividing cells, and up to 15 months using DNMT3A/L with KRAB (Nuñez et al., 2021; O’geen et al., 2022). This greatly increases the therapeutic viability of such systems as they will have a prolonged effect, which could be even persistent in non-dividing neurons as the epigenetics marks will not be under the pressure of mitosis. Additionally, as these therapeutics target endogenous genes, they are more likely to keep gene expression within a physiologically relevant window making them a more attractive approach than RNAi or overexpression cassettes that may reach supraphysiological levels (Lorsch et al., 2019; Peter et al., 2019; Lu et al., 2020). This makes epigenetic editing a promising approach for many neurological disorders including Fragile X/FXTAS, Huntington’s Disease, Spinocerebellar Ataxias, Alzheimer’s Disease, Parkinson’s Disease, and other disorders characterized by neuronal cell death (Table 1).

**TABLE 1:** Current approaches for therapeutic development in dominant neurodegenerative disorders.

Disorder	Gene	Current Approaches	Clinical trials (Y/N)	References
Huntington’s Disease (HD)	*HTT*	ASO	Y	ClinicalTrials.gov ID NCT02519036, NCT05032196
		miRNA	Y	ClinicalTrials.gov ID NCT04120493
		Zinc Finger-KRAB	N	Zeitler et al. (2019)
Spinocerebellar Ataxia type 2 (SCA1)	*ATXN1*	ASO	N	O’Callaghan et al. (2020)
Spinocerebellar Ataxia type 2 (SCA2)	*ATXN2*	ASO	N	Scoles et al. (2017)
		ASO	Y	ClinicalTrials.gov ID NCT04494256
Spinocerebellar Ataxia type 2 (SCA3)	*ATXN3*	ASO	N	Moore et al. (2017)
Spinocerebellar Ataxia type 7 (SCA7)	*ATXN7*	ASO	N	Niu et al. (2018)
		RNAi (Mirtrons)	N	Curtis et al. (2017)
Alzheimer’s Disease (AD)	*APOE*	ASO	N	Huynh et al. (2017)
Parkinson’s Disease (PD)	*SNCA*	dCas9-DNMT3A	N	Kantor et al. (2018)
	*LRRK2*	ASO	N	Zhao et al. (2017)
Neurodegeneration	*BDNF*	CRISPRa	N	Savell et al. (2019)
	*GDNF*	Zinc Finger	N	Laganiere et al. (2010)

ASO, Antisense oligonucleotide; CRISPRa, CRISPR activation.

### Fragile X/FXTAS

Fragile X is the most common form of a single-gene autism spectrum disorder (Kaplan and McCracken, 2012). Fragile X is caused by a CGG repeat in the 5′ UTR of the *FMR1* gene. This CCG expansion is unmethylated until it reaches above 200 repeats, where the gene becomes hypermethylated and is no longer expressed. Persistent demethylation of *FMR1* in Fragile X iPSCs via lentiviral transduction of a dCas9-Tet1 fusion was able to show the rescue of electrophysiological abnormalities when differentiated into neurons (Liu et al., 2018). While this approach shows promise, there is also some concern about gene reactivation as there is a premutation in the FMR1 gene, where 55-200 CGG repeats do not induce hypermethylation but cause Fragile X-associated tremor/Ataxia Syndrome (FXTAS). FXTAS is a degenerative disorder where the *FMR1* CGG expansion leads to toxic RNA species that lead to a variety of phenotypes, including cerebellar gait ataxia, intention tremor, frontal executive dysfunction, and global brain atrophy (Leehey, 2009). FXTAS is an interesting candidate for epigenetic downregulation, as reducing *FMR1* expression may hinder the effect of the toxic RNA species, but total knockdown of *FMR1* will likely be deleterious as seen in Fragile X. The opposing pathology of toxic RNA in FXTAS but haploinsufficiency seen in Fragile X presents the possibility for a two-pronged approach of epigenetic editing to downregulate the mutant gene, along with the addition of gene therapy to deliver copies of the unexpanded *FMR1* gene to prevent the consequences of haploinsufficiency of FMR1.

### Huntington’s disease and the case for allele specificity using dCas9

Huntington’s Disease is a quintessential dominant disorder that is very amenable to silencing as a therapeutic approach. Huntington’s Disease (HD) is caused by a trinucleotide repeat in exon 1 of the Huntingtin (*HTT*) gene. While the full function of HTT has not been elucidated, it is known to be important in development and is involved in RNA trafficking, vesicle transport, and transcriptional regulation (Schulte and Littleton, 2011). In contrast to loss-of-function mutations, GOF proteins often still retain much of their normal function and their protein structure is less destabilized overall (Gerasimavicius et al., 2022). In Huntington’s disease, complete knockouts of *HTT* are embryonic lethal, whereas age-of-onset and life expectancy in rare cases of patients with two expanded alleles are similar to those with only one expanded gene copy (Dragatsis et al., 1998; Cubo et al., 2019).

This leads us to believe that even bearing the gain-of-function mutations, the mutant HTT protein is still able to perform some of its necessary functions, which may make total knockdown deleterious. A unique feature of *HTT* is that it is highly conserved across species and contains haplotype blocks that have SNPs that segregate with the expanded allele (Warby et al., 2011). This allows for allele-specific gene regulation through the targeting of SNPs associated with the expanded allele. These alleles are also found at high frequencies within the patient population, increasing the viability of this approach. Within the last 4 years, 3 clinical trials were using an ASO approach, including one using an SNP targeting approach for allele specificity, demonstrating its clinical feasibility (Schulte and Littleton, 2011).

CRISPR-dCas9 allows for a variety of ways to modify allele specificity on both the dCas9 level as well as the sgRNA level. Allelic discrimination is usually determined by single nucleotide polymorphisms (SNPs) in the target sequence that can be used to bind to one allele over the other (Christie et al., 2020). sgRNA specificity can be changed based on mismatch tolerability, as mismatches within the seed region (bases 1–10 proximal to the PAM site) tend to have a greater effect on binding efficiency (Hsu et al., 2013; Doench et al., 2016). Adding in additional mismatches may further allow for allelic discrimination by decreasing binding to the non-targeted allele (Kim et al., 2014). It is also possible to titer sgRNA amounts to decrease off-target binding, which may also help with allele discrimination (Hsu et al., 2013). There are also some groups working to use chemical modification to increase sgRNA specificity, such as 2′-O-methyl-3′-phosphonoacetate, but this approach is not viable for many viral or plasmid-based delivery modalities that require transcription of the sgRNA in the target cell (Hu et al., 2018). Adding a secondary structure, such as hairpin loops, to the spacer sequence of the sgRNA has also been shown to improve allele discrimination (Kocak et al., 2019). It is also possible to use different dCas9 variants to increase binding efficiency. Similar to the sgRNA, decreasing the amount of dCas9 protein in the cell can help with allele specificity and reduce off-targets. One way to do this is by delivering the dCas9 as either mRNA or as a ribonucleoprotein complex rather than as plasmid or DNA to reduce the total number of copies within the cell (Ramakrishna et al., 2014; Miller et al., 2017). CRISPR-Cas9 knockout approaches have shown the ability to reduce some HD transcriptome dysfunction *in vitro* and motor phenotypes *in vivo* but have not translated to clinical trials, as allele and gene selectivity when targeting a repeat is difficult (Becanovic et al., 2015; Xu et al., 2017; Ikeda et al., 2020). This, along with the fact there are well-characterized mutations in transcription factor binding sites in the *HTT* promoter that affect age of onset make epigenetic downregulation not only a promising approach but possibly more robust as dCas9 binding will likely induce further downregulation through the blocking of these transcription sites (Becanovic et al., 2015).

### Spinocerebellar ataxias

Spinocerebellar ataxias (SCAs) also present a way to target a group of disorders caused by different genes using similar therapeutic approaches. Spinocerebellar ataxia (SCAs) is a group of progressive disorders that are characterized by a lack of coordination that is thought to be due to degeneration and dysfunction of the cerebellum and its associated pathways. There are over 40 genetically distinct SCAs, including 12 repeat expansion SCAs, 6 of which are caused by a CAG repeat expansion. While RNA knockdown approaches are a promising therapeutic for some SCAs, there are several SCAs (SCA10, SCA31, SCA36, and SCA37) that are known to have RNA toxicity-induced pathogenesis, making a DNA targeting approach preferable (Scoles et al., 2017; Swinnen et al., 2020). Some groups have investigated a silence and replace strategy for SCA7 by downregulating the causative mutation by RNAi and delivering a functional copy of the gene *in vitro*, but the delivery of this modality and the regulation of gene expression is not fully elucidated (Curtis et al., 2017). Epigenetic downregulation presents a promising approach that could allow for a single platform to treat a multitude of disorders by simply changing the sgRNA for the implicated SCA gene.

### Alzheimer’s and Parkinson’s

It is also possible to begin looking at epigenetic editing for more common disorders that have known genetic links. Hereditary autosomal dominant forms of Alzheimer’s Disease (AD) and Parkinson’s Disease (PD) are associated with mutations in *APOE*, as well as mutations in *SNCA* and *LRRK2,* respectively. Several groups have shown lowering of *APOE* variant *APOE4*, whether by converting it to a non-pathogenic *APOE3* through CRISPR-Cas9 or by lowering expression through an ASO can reduce Aß plaques (Huynh et al., 2017; Lin et al., 2018). While these approaches show promise, the ASO approach was only able to decrease Aβ with treatments at early time points, and the cells that were treated with CRISPR-Cas9 to convert *APOE4* to *APOE3* still had high levels of Aβ_42_ secretion from the treated cells. CRISPR-dCas9 epigenetic editing may prove to be a more potent approach as it is often more efficient than Cas9 gene editing and may be more robust at later time points than ASOs. *SNCA* has been silenced through a shRNA, but it did not fully protect from dopamine neuronal loss (Khodr et al., 2011). *SNCA* has also been downregulated through Cas9 gene editing (Yoon et al., 2022). Both the shRNA and CRISPR-Cas9 approaches used a viral overexpression model of a causative *SNCA* mutation, making the clinical relevance of the findings less clear, as they do not target the endogenous gene and the pathogenesis seen using a viral overexpression model may not accurately recapitulate disease state. Some groups have used dCas9 epigenome editing to downregulate the endogenous *SNCA* via DNMT3a DNA methylation, which showed increased cell viability and reduction of mitochondria-associated superoxide production *in vitro* (Kantor et al., 2018). *LRRK2* has been targeted with ASOs for limited success *in vitro* and *in vivo*, showing the clinical feasibility of an *LRRK2* depletion strategy (Zhao et al., 2017). Some groups have also tried completely removing the *LRRK2* gene via zinc finger nucleases, and although this is not a clinically favorable approach due to the difficulties with *in vivo* gene editing, it does show some rescue of phenotypes in cultured neurons, indicating a knockdown approach would be beneficial (Reinhardt et al., 2013). *APOE4*, *SNCA*, and *LRRK2* are all ideal targets using a dCas9 epigenetic-based approach, as there is clear pathogenesis that can be ameliorated through modulation of gene expression. This opens the field of possibilities for those suffering from hereditary forms of AD and PD.

### Upregulation of neuroprotective genes

There is also the possibility of using epigenetic editing to upregulate neuroprotective genes, which could apply to a multitude of neurodegenerative disorders. Epigenetic editing via TALEs, zinc fingers, and dCas9 can be used to upregulate genes in haploinsufficiency, or X-linked disorders (Halmai et al., 2020; Deng et al., 2022) but is not ideal for disorders caused by a toxic protein, as most autosomal dominant disorders are. While gain-of-function mutations are not good targets for upregulation, many diseases have known genetic modifiers that reduce disease progression or age-of-onset. This presents an additional approach that can be used in combination with the targeting of the causative mutation through downregulation, or as an alternative when the causative mutation is not amenable to downregulation. Many genes are known to be neuroprotective, such as brain-derived neurotrophic factor (*BDNF*) and glial cell line-derived neurotrophic factor (*GDNF*), which could be wildly applicable as gene therapy for many neurological disorders that occur due to a decrease in neuron health and increased cell death (Figure 3) (Laganiere et al., 2010; Nagahara and Tuszynski, 2011).

The most widely used effector domain for the upregulation of gene expression is VP64, which consists of 4 VP16 domains that recruit various transcriptional activators to facilitate the assembly of the pre-initiation complex (Beerli et al., 1998; Hirai et al., 2010; Maeder et al., 2013; Perez-Pinera et al., 2013). Recently, many groups have moved towards the use of multiple effector domains to achieve higher levels of upregulation. A tripartite activator VP64-p65-Rta (VPR) has been increasingly used as it shows more robust upregulation of gene expression compared to a single VP64 alone with less sgRNA multiplexing required to observe gene modulation (Chavez et al., 2015). Another addition to the CRISPR-dCas9 system to increase gene expression is SunTag. SunTag is a polypeptide chain that can recruit epitopes derived from the single-chain variable fragment (scFv) that can then recruit additional VP64 effectors (Tanenbaum et al., 2014). This allows for the recruitment of 10 or 24 VP64s to the same target site, greatly improving the efficacy of upregulation. Another 3 component system that can be used to aid in gene upregulation is the synergistic activation mediator (SAM) system. SAM primarily works through the addition of aptamers to the sgRNA scaffold that allows for the recruitment of MS2 proteins that are fused to additional effectors (Konermann et al., 2015). This has rapidly improved the ability to use epigenetic editing to upregulate genes, making the prospect of its use to increase the expression of protective genes even more feasible therapeutically.

## Delivery of dCas9 to the CNS

One major barrier to moving these epigenetic editor tools to the clinic is the delivery of these large proteins to the brain. When determining which delivery modality to use, packaging capacity, transience, tropism, and size of the brain region being targeted are important caveats. dCas9 alone is approximately 3.5kb, making it too large to fit into most clinically applicable viral vectors, like adeno-associated virus (AAV) with the addition of a strong promoter and effector domains. Many groups are working to circumvent this through novel delivery modalities, including using a split dCas9 AAV platform. This involves separating the dCas9 into two lobes and fusing each lobe to an intein that will then trans-splice and produce a full-length dCas9 when in target cells (Truong et al., 2015). While AAV has a strong clinical profile and is used in several FDA-approved therapies, the dual AAV system does decrease efficacy as a cell must be infected by both viruses to elicit the desired biological effect.

Other groups are looking to transient delivery systems that deliver either the Cas9 protein itself or the mRNA encoding Cas9. Many groups have worked to deliver the Cas9/sgRNA ribonucleoprotein complexes (RNPs) using cell-penetrating peptides (CPPs) that are fused directly to the Cas9 protein and allow the RNP to permeate the cell membrane (Ruseska and Zimmer, 2020; Gustafsson et al., 2021). CPPs have also been tested minimally *in vivo* and likely have low efficacy due to their poor stability, limited cellular uptake, and poor target specificity.

Lipid nanoparticles (LNPs) consist of ionizable cationic lipids, cholesterol, amphipathic phospholipids, and poly(ethylene glycol) lipids that encapsulate the protein or mRNA of interest and enter the cell through membrane fusion or receptor-mediated endocytosis (Li and Szoka, 2007). LNPs have become increasingly popular due to their use in the COVID-19 vaccines and have long been used for Cas9 delivery (O’geen et al., 2022). LNPs have a virtually unlimited packaging capacity, making them a strong candidate for epigenetic editors as the fusion of effectors domains greatly increases the size capacity needed. Several groups have worked to improve the ability to target different tissues by modulating the composition of the LNPs by changing the ratios of the lipid types or through the addition of specific lipids that have an affinity for different tissues (Cheng et al., 2020). While the immunogenicity of LNPs is low and allows for redosing, the LNPs have a natural affinity for targeting the liver and the ability to target the brain is low and needs improvement (Kenjo et al., 2021).

A similar technology based on a viral packaging system termed viral-like particles (VLP) has been gaining popularity in recent years (Mangeot et al., 2019; Gee et al., 2020; Banskota et al., 2022). Direct fusion of the viral packaging protein gag to Cas9 allows for packaging into extracellular vesicles without the packaging of a viral genome (Mangeot et al., 2019; Banskota et al., 2022). VLPs also have a unique ability pseudotyped to modulate tropism and the tissue targeted (Hamilton et al., 2021). Due to the novelty of VLP, it requires further testing to understand its applicability as a therapeutic platform, as its immunological effect and the ability to scale to clinically relevant doses is unclear.

While several delivery modalities show promise, none have been able to show widespread effects therapeutically using dCas9 for multiple CNS indications at this point and which modality should be used varies greatly on the disease and cargo being delivered.

## Discussion

The field of epigenetic editing is rapidly growing and has large therapeutic potential, particularly when it comes to dominant neurological disorders. Many neurological disorders are caused by aberrant gene expression, making them uniquely apt for transcriptional regulation-based therapeutics. TALEs, zinc fingers, and dCas9 can be used as DNA binding domains fused to effectors domains to hypermethylate promoters and remodel chromatin to downregulate gene expression. These tools have the potential to be used in the clinic for both the targeting of causative mutations, as well as the targeting of known genetic modifiers of disease state to alleviate symptoms and disease pathology in numerous dominant neurological disorders. Dominant disorders have been especially hard to target due to their gain-of-function pathology, making the epigenetic editing approach encompass high therapeutic value. Although there are many promises of epigenetic editing as a therapeutic, there are still many obstacles to overcome when moving this technology into the clinic.

Another important note is the assessment of off-target effects. While there is a less off-target burden using an epigenetic editing system compared to a nuclease, there still needs to be an in-depth understanding of off-target potential before moving into the therapeutic realm. Although it is not necessary to assess genomic alterations, understanding the changing epigenetic landscape after treatment with these constructs is important. Looking at not only localized changes but genome-wide changes to histone tail modifications along with DNA methylation changes will be an important marker of the specificity of treatment. It should also be noted that it is important to understand dCas9 binding genome-wide for each change in sgRNA, as that can modify off-target potential. Finally, it will be important to assess changes to the transcriptome, as that is the most likely and meaningful off-target from these epigenetic editors.

Another consideration that is very pertinent for neurological disorders is the critical window for treatment. Neurodevelopmental disorders are often diagnosed after the onset of symptoms or lack of reaching developmental milestones. Depending on the disease severity and phenotype, this can range from several days after birth to several years, which alters the feasibility of early treatment. Many neurodevelopmental disorders are characterized by large-scale changes to regions of the brain, which may be incapable of changing even after treatment. While the brain retains much plasticity throughout childhood, it is unknown whether treatment after birth or possibly *in utero* would be able to have large-scale effects. Neurodegenerative disorders present a similar but inverse problem, as many of these diseases are not diagnosed until there is large-scale neuronal death and dysfunction. One promising idea that has emerged from the HD field is the idea of the “Huntingtin Holiday,” where treatment may be able to not only stop the decline of a disorder but improve symptoms by rescuing dysfunctional cells that were nearing cell death (Lu and Yang, 2012). While this would not replace cells that were lost during the disease progression, it could still alter the disease state and improve symptoms.

In this review, we discuss the emerging field of epigenetic editing and its application to dominant neurological disorders. The field is rapidly evolving with different dCas9 variants, effector domains, and sgRNA modification strategies, as well as a deeper understanding of the pathogenesis of many dominant disorders. While there are many improvements to be made in the field to bring this approach to the clinic, epigenetic editing has a strong therapeutic potential for those suffering from dominant neurological disorders.
